# Surgical Management of Hidradenitis Suppurativa Using Staged Carbon Dioxide Laser Marsupialization

**DOI:** 10.3390/jcm15176653

**Published:** 2026-08-28

**Authors:** Sydney R. Resnik, Alexander R. Gomez-Lara, Lauren Fernandez, Irena Pastar, Akhil Wadhera, Paul G. Hazen, Barry I. Resnik

**Affiliations:** 1 Dr. Phillip Frost Department of Dermatology and Cutaneous Surgery, Miller School of Medicine, University of Miami, Miami, FL 33125, USA; lif28@miami.edu (L.F.); ipastar@med.miami.edu (I.P.); bresnikmd@gmail.com (B.I.R.); 2 Resnik Skin Institute, Aventura, FL 33180, USA; 3 Kaiser Permanente Bernard J. Tyson School of Medicine, Pasadena, CA 91101, USA; alex.gomezlara@kp.org; 4 Center for Laser Surgery, Kaiser Permanente, Union City, CA 94612, USA; akhil.wadhera@kp.org; 5 Department of Dermatology, University Hospitals of Cleveland, Cleveland, OH 44106, USA; paulhazen@aol.com; 6 Hidradenitis Suppurativa Institute, Associates in Dermatology, Westlake, OH 44145, USA

**Keywords:** hidradenitis suppurativa, staged carbon dioxide laser marsupialization, wound healing, recurrence, office procedures

## Abstract

**Background**: Hidradenitis suppurativa (HS) is a chronic disease marked by symptoms of pain, drainage, and odor. Persistent abscesses, nodules, and scarred tunnels can be resistant to pharmacologic management, with chronic lesions often requiring complex surgical intervention. Treatments include en bloc excision with primary closure, tissue flaps or skin grafts. Such procedures are associated with post-operative challenges, including pain, infection, contractures, and high recurrence rates. **Methods**: Herein we report use of continuous-wave carbon dioxide (CO_2_) laser for HS tissue removal, during which there is an initial full-thickness excision of clinically evident lesions of hidradenitis, followed by the intra-surgical identification and staged removal of additional areas, resulting in a pocket-like (marsupialized) defect. We named the procedure staged carbon dioxide laser marsupialization (SCLM). **Results**: Five hundred twenty-seven patients underwent a total of 992 laser surgery sessions to manage 1846 treatment sites using SCLM. All but six patients were Hurley Stage II or III. The commonly treated sites included the groin (29.4%), axillae (27%), and buttocks (10.9%). A histomorphometry analysis of a subset of tissues revealed an average depth of HS tunnels of 3.34 mm. All post-SCLM wounds healed by secondary intention, with full healing achieved in 6–16 weeks. No instances of post-operative infection occurred. Documented surgical-site recurrence occurred in 1.4% of treated sites during available follow-up. **Conclusions**: Staged carbon dioxide laser marsupialization for removal of lesions of HS is an encouraging treatment approach for patients with moderate-to-severe HS, with an excellent quality of healing, no infection risk, minimal post-surgical complications, and low frequency of documented surgical-site recurrence.

## 1. Introduction

Hidradenitis suppurativa (HS) is a chronic, recurrent inflammatory skin disease characterized by persistent and painful cutaneous nodules, abscesses, draining tunnels, and progressive scarring [1]. Disease involvement most commonly affects intertriginous regions, including the axillary, anogenital, inguinal, and perianal/gluteal areas. Although the pathogenesis of HS remains incompletely understood, follicular occlusion and rupture with subsequent dysregulation of innate and adaptive immune responses are thought to contribute to persistent inflammation and tissue destruction [2]. In more advanced disease, chronic inflammation may result in epithelialized tunnels and extensive fibrosis, producing structural changes that persist despite control of more superficial inflammatory activity [3].

The burden of HS extends beyond its visible cutaneous manifestations. Pain is among the most prominent symptoms [4], while recurrent drainage, malodor, scarring, and involvement of intimate anatomical sites may interfere with mobility, sleep, sexual activity, interpersonal relationships, and activities of daily living [4,5]. HS is associated with substantial psychosocial morbidity, and may significantly impair occupational functioning and work productivity [6]. The recurrent and unpredictable nature of the disease can further compound these effects. Consequently, HS has a profound impact on quality of life, particularly among patients with severe or extensively involved disease.

Management of HS is guided by disease severity, inflammatory activity, anatomic involvement, and the presence of irreversible structural disease. Medical treatment may include topical and systemic antibiotics, hormonal therapies, retinoids, and targeted systemic immunomodulatory therapy [1,2]. The introduction of biologic therapies has substantially expanded the treatment options for patients with moderate-to-severe HS. However, medical therapies primarily target inflammatory disease activity and may not adequately address established epithelialized tunnels, fibrosis, and chronically scarred plaques. Accordingly, in patients with severe HS, presence of structurally advanced disease decreases their likelihood of durable response to pharmacologic therapies alone [3]. For such patients, surgical intervention is frequently required in addition to medical management.

Several surgical approaches have been utilized in HS. The most common first-line technique is incision and drainage. This is often used for acutely inflamed abscesses and may provide symptomatic relief; however, recurrence rates are high and the underlying HS tunnels are not addressed [7]. Deroofing is an alternative technique that removes the skin covering the roof of nodules, abscesses, or sinus tracts, followed by drainage and curettage of the edges. The base is allowed to remain intact [7], and healing is by secondary intention, with high recurrence rates [7]. Scalpel excision of solitary lesions, or wide local excision for more extensive disease, is most often employed for persistent or extensive lesions, especially those associated with scarring. However, again, recurrence is common, with rates ranging from 18 to 54% [8,9].

Carbon dioxide (CO_2_) laser surgery provides an alternative approach to removal of advanced HS lesions [10]. A CO_2_ laser utilizes both focused cutting and ablative methods, providing simultaneous tissue removal and hemostasis. In contrast to conventional excision techniques, CO_2_ laser marsupialization permits controlled removal of clinically apparent disease followed by the sequential intra-operative identification of sinus tract extensions using probe-guided delineation [7]. Marsupialization followed by laser ablation facilitates greater visualization due to excellent hemostasis and facilitates targeted removal of interconnected tracts during the same operative session, with subsequent healing by secondary intention.

We herein report on a large, multicenter study including 527 patients who underwent 992 surgical sessions for treatment of 1846 individual HS sites using staged CO_2_ laser marsupialization (SCLM). We evaluated the surgical-site recurrence, post-operative infection, complication rates, and wound healing across the anatomically complex regions commonly affected by moderate-to-severe HS.

## 2. Materials and Methods

### 2.1. Study Design

The study was retrospective and included patients who underwent SCLM for HS at three independent clinical centers:

1. Center 1: Hidradenitis Suppurativa Institute, Cleveland, OH, USA;
2. Center 2: Resnik Skin Institute, Aventura, FL, USA;
3. Center 3: Center for Laser Surgery, Kaiser Permanente, Union City, CA, USA.

Patients were selected for SCLM based on the presence of persistent abscesses, nodules, and/or scarred tunneled plaques. Patients were not required to have controlled disease, to be receiving medical therapy, or to have failed pharmacologic treatment prior to surgery. There are no absolute contraindications to undergoing SCLM. There was no body mass index (BMI) restriction. Pregnancy was considered a relative contraindication, with surgery generally deferred unless deemed clinically necessary. The pre-surgical evaluations included assessment of HS severity and distribution, patient demographics, comorbidities, and current and prior medical therapies. The defining features of the surgery, as well as surgical complications, risk of recurrence, and patient acceptance were also determined. The combined database across the three centers included 527 patients who underwent SCLM between January 1981 and December 2024.

### 2.2. Surgical Technique

All patients received surgery using a continuous-wave CO_2_ laser (Center 1: Lumenis Sharplan 40C; Center 2: Lumenis Sharplan 1100; Center 3: Lumenis Ultrapulse) with a spot size of 0.22 or 1 mm and an energy setting between 15 and 40 watts. In most cases, office-based local or tumescent anesthesia [11], using lidocaine with or without epinephrine, was used. General anesthesia within an ambulatory surgical facility was utilized in 92 cases.

In all instances, the planned surgical boundaries were defined on clinical grounds as determined by visual inspection and palpation. The laser was used in continuous-wave cutting mode to remove the principal, clinically identified inflammatory masses with a 2–5 mm margin. This technique was applied for each type of inflammatory lesion: nodule, abscess, or scarred tunnel plaques. Their removal was designed to identify and subsequently dissect the inflammatory processes from the lateral and deep normal tissue, while sparing normal contiguous tissue.

### 2.3. Tissue Specimens

The protocol for tissue collection was approved by the Institutional Review Board (IRB) at the University of Miami (protocol #20200187). Informed consent was obtained from patients (*n* = 15; mean age ± standard deviation = 31.7 ± 9.5; 9 females, 6 males) diagnosed with HS tunnels and/or their legal guardian(s) after discussion of research prior to the procedure, to allow for any concerns or questions to be addressed. Skin specimens were collected from the discarded tissue after excision at Center 2 to analyze the tissue pathology.

### 2.4. Histology Imaging and Measurements

The specimens were fixed in formalin and processed for paraffin embedding and stained with Hematoxylin and Eosin (H&E) to assess the tissue morphology, as previously described [12]. A microtome was used to cut the tissue into five micrometer sections following H&E staining [13]. The histology was analyzed with an Olympus Slide Scanner microscope, and digital images were obtained using OlyVIA Olympus software (version 2.9). The depth of the tunnel was measured in micrometers from the epidermis to the epithelial layer of the tunnel using ImageJ software (version 2.14.0/1.54f).

### 2.5. Data Analysis

Given the retrospective nature of this study, the data analyses are primarily descriptive. The continuous variables are reported as the mean ± standard deviation (SD) and range where applicable. The categorical variables are summarized using frequencies and percentages. The surgical outcomes, including documented recurrence and post-operative complications, are reported as counts and proportions of treated sites or patients, as appropriate. No formal hypothesis testing or comparative inferential analyses was performed.

## 3. Results

### 3.1. Patient Demographics Revealed High Prevalence of Acne, Pilonidal Sinus, Family History of HS, and Obesity

A total of 527 patients were treated at three independent clinical centers for the treatment of HS. Three hundred sixty-six females and 161 males underwent 992 surgical sessions for treatment of 1846 affected sites (Table 1). At Center 1, 292 patients underwent a total of 671 surgeries, with 1179 treated areas. At Center 2, 181 patients underwent a total of 253 surgeries with 592 sites treated. At Center 3, 54 patients underwent a total of 68 surgeries with 75 sites treated (Table 2).

Across all sites, most patients were Hurley Stage III (overall 72.5%), and the mean Hurley Stage among the CO_2_ laser-treated patients was 2.6. Each individual was pre-surgically evaluated for potential operative risks and comorbidities. The comorbidity data included obesity (average BMI 31.5); smoking (22.6%); family history of HS (32.6%); depression (12.1%); and the presence of pilonidal sinus (39.6%), acne (44.8%), or folliculitis decalvans (11.3%) (Table 1).

### 3.2. SCLM Resulted in Complete Removal of Affected Tissue and Minimal Bleeding

The surgical sites generally represented the most severe and/or symptomatic areas. Most procedures were performed using local anesthesia in an office setting. General anesthesia was utilized in 92 of the 527 patients (17.4%), typically for extensive or anatomically complex disease. In patients with multiple involved areas, 1–3 anatomic sites were treated per surgical session. The distribution of areas treated is presented in Table 2, with the most common locations being the groin (including inguinal folds, vulva, and labia)—542 sites (29.4%); axilla—498 sites (27.0%); and buttocks—201 sites (10.9%).

**Figure 1:**
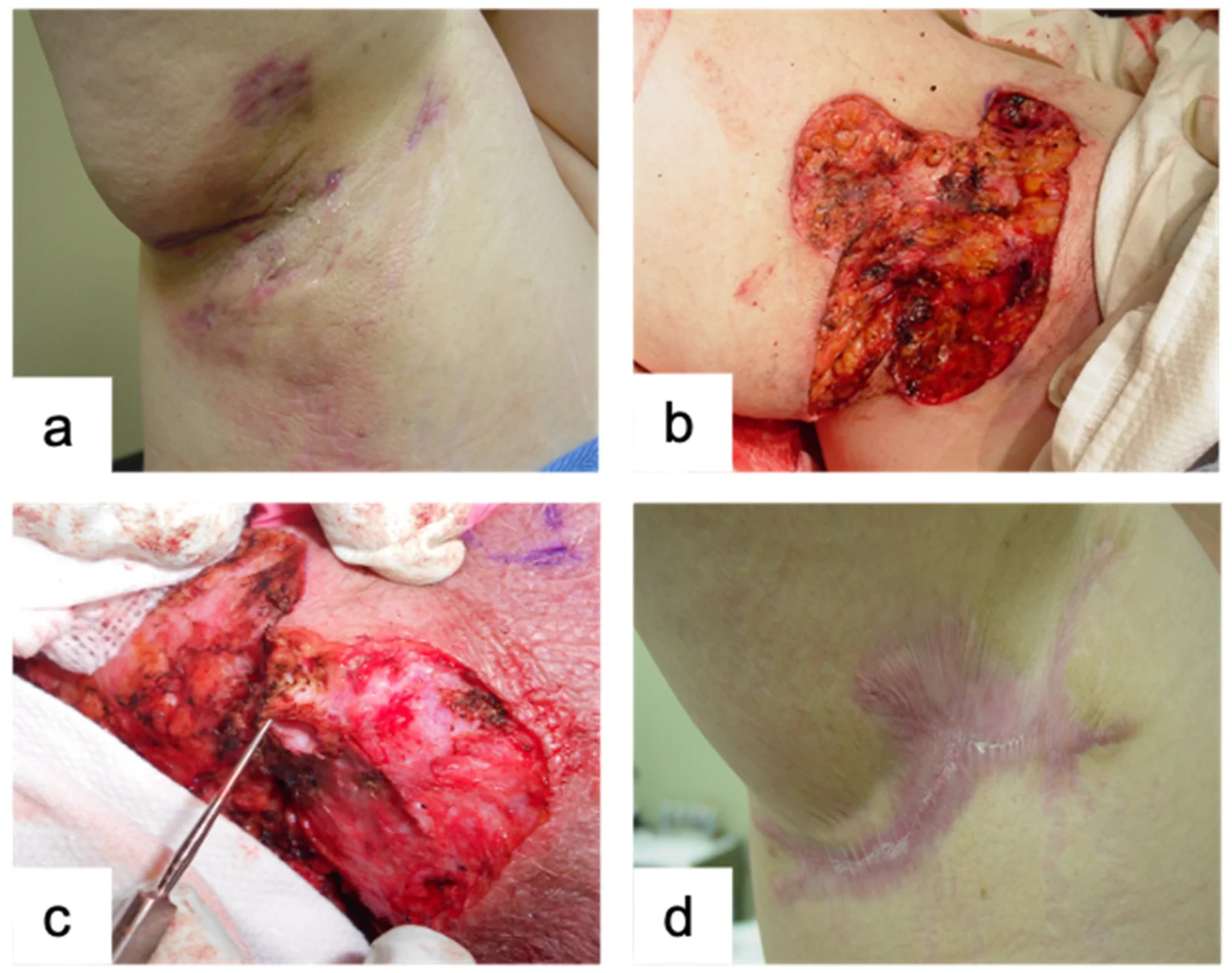
Representative case of axillary Hurley Stage III plaque treated with SCLM. (**a**) Pre-operative: right axilla, Hurley Stage III; (**b**) post-operative: immediately following CO_2_ laser marsupialization; (**c**) intra-operative identification of sinus tract; (**d**) three months post-operative.

**Figure 2:**
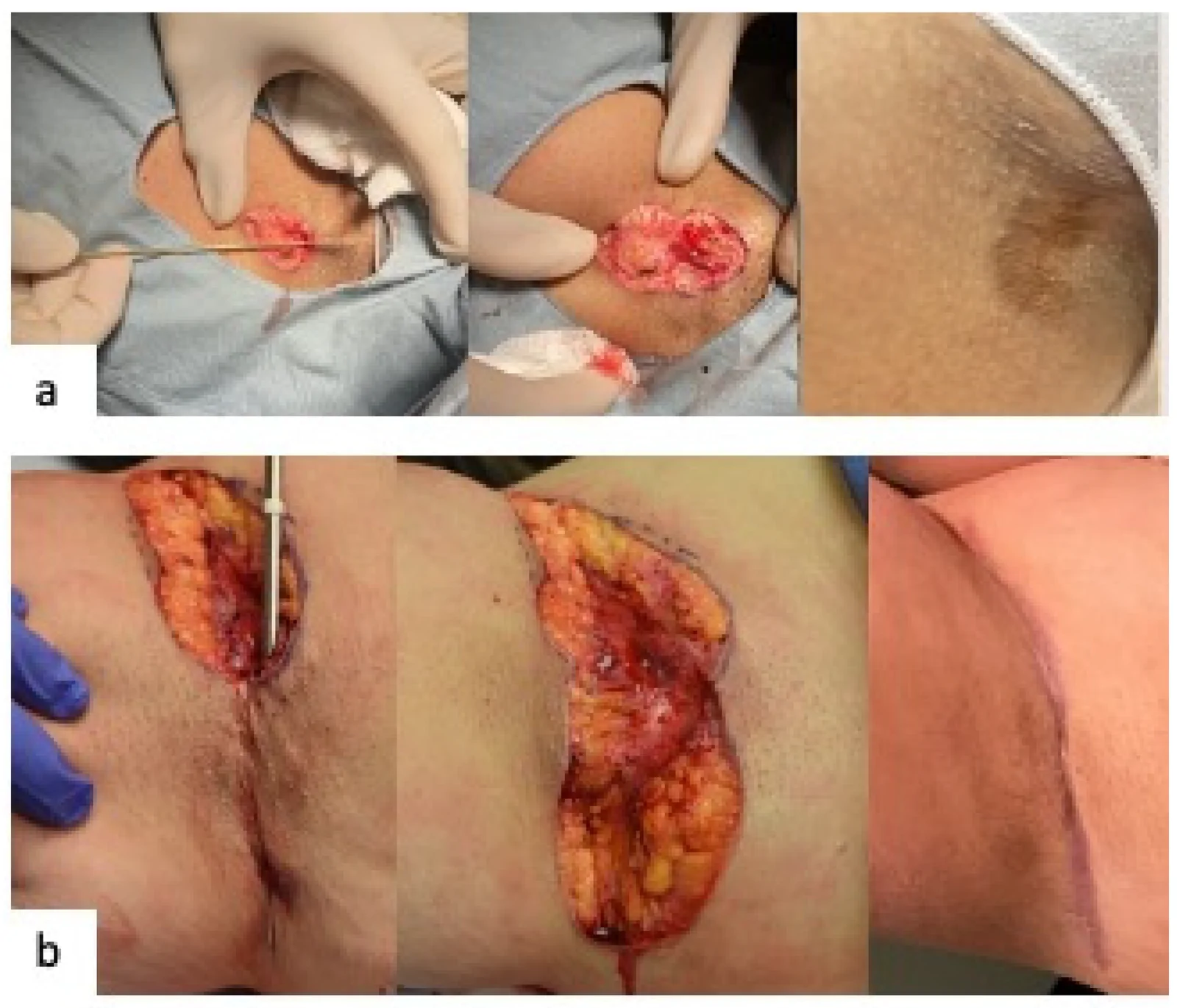
Use of metal probe intra-operatively for identification of tunnel extension. (**a**) Right inguinal fold, intra-operative identification of tunnel. (**b**) Right axilla, intra-operative identification of tunnel; note the metal probe extending out of the tunnel at the inferior side.

**Figure 3:**
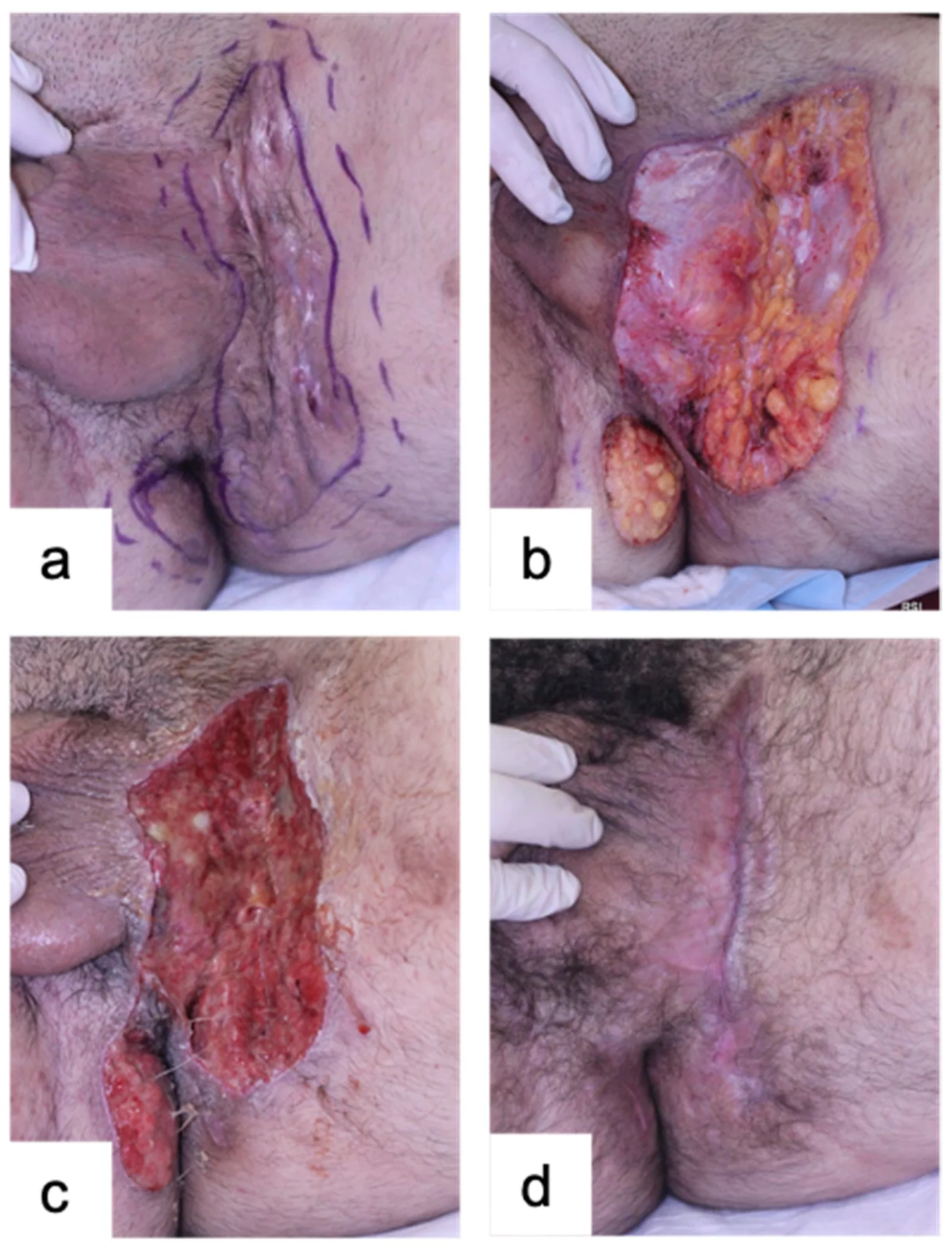
Representative case of inguinal Hurley stage III plaque treated with SCLM. (**a**) Pre-operative: left inguinal fold, Hurley Stage III; (**b**) post-operative: immediately following CO_2_ laser marsupialization; (**c**) post-operative Day 8; (**d**) three months post-operative.

The goal was complete removal of affected tissue, normally to the level of the subcutis. Following initial removal, extensions and occult cavitary areas (Figure 1 and Figure 2) were identified using a metal probe within the surgical defect or by direct visualization. Using the laser in focused mode, additional stages of full-thickness removalsof involved skin were carried out, until the entire surgical field was free of extensions. Where needed, persistent bleeding was controlled with the laser, spot electrocautery, or placement of interrupted 3-0 Vicryl sutures. In each case, the removal resulted in a pocket-like (marsupialized) defect with subcutaneous tissue at the base and normal tissue at the lateral margins of the surgical defect. To complete the procedure, the CO_2_ laser was used in defocused/scanning mode to vaporize the base and margins of the wound and to produce a charred surface, a process we termed “carmelization”, thereby creating a minimally bleeding field and a uniform plane for healing. For some patients with large areas of HS, it was decided to perform only partial removal of extensive areas given the dose limitations of local anesthesia, as well as the goals of comfortable post-operative management of the surgical defect and preservation of normal life activities. After at least 2 months, additional surgeries were an option to address adjacent untreated areas of disease. All areas healed by secondary intention (Figure 1 and Figure 3).

All patients were instructed to continue their current courses of therapy, including antibiotics and/or biologic agents during and after the surgery. One hundred one patients (19.2%) had a history of previous or current treatment with biologics, with adalimumab (60) and infliximab (19) being the most used (Table 2).

### 3.3. Surgical Site Recurrence Rates Post-SCLM Were Low

The healing time, post-operative complications, and recurrence rates were evaluated by follow-up office visits and/or phone communications. The healing times were proportional to the defect sizes, and ranged from 6 to 12 weeks for smaller wounds, and up to 16 weeks for larger defects (Figure 1 and Figure 3). Wound healing was managed daily using warm tap water compresses (Centers 1 and 2) and/or pure hypochlorous acid 0.033% (Center 3), followed by Vaseline or Medi-Honey applications. Post-operative pain management was provided as clinically indicated, including topical lidocaine 2% ointment or short courses of systemic opioid analgesia when needed. Non-stick pads and gauze dressings were used for daily wound care. All areas healed by secondary intention, and no post-operative infection was reported. Post-operative bleeding requiring hospitalization was recorded for two patients and with four occurrences. One of these two patients was on antiplatelet medication, which likely contributed to the three episodes of bleeding following three separate surgeries.

Surgical-site HS recurrence was defined as evidence of post-surgical papules, nodules, or tunnels within or abutting the treated area or within 2 mm of the surgical scar. Recurrence was assessed through available follow-up office visits and/or telephone communications. Duration of documented follow-up varied among patients, ranging from 4 months to 25 years. Recurrences were documented in 17 of 1179 treated sites at Center 1, eight of 592 sites at Center 2, and 0 of 75 sites at Center 3, corresponding to 25 of 1846 treated sites (1.4%). For analytic purposes, the sites without documented recurrence during the available follow-up period were classified as non-recurrent.

### 3.4. Histopathological Analyses Revealed Significant Depth of Epithelialized Tunnels Post-SCLM

**Figure 4:**
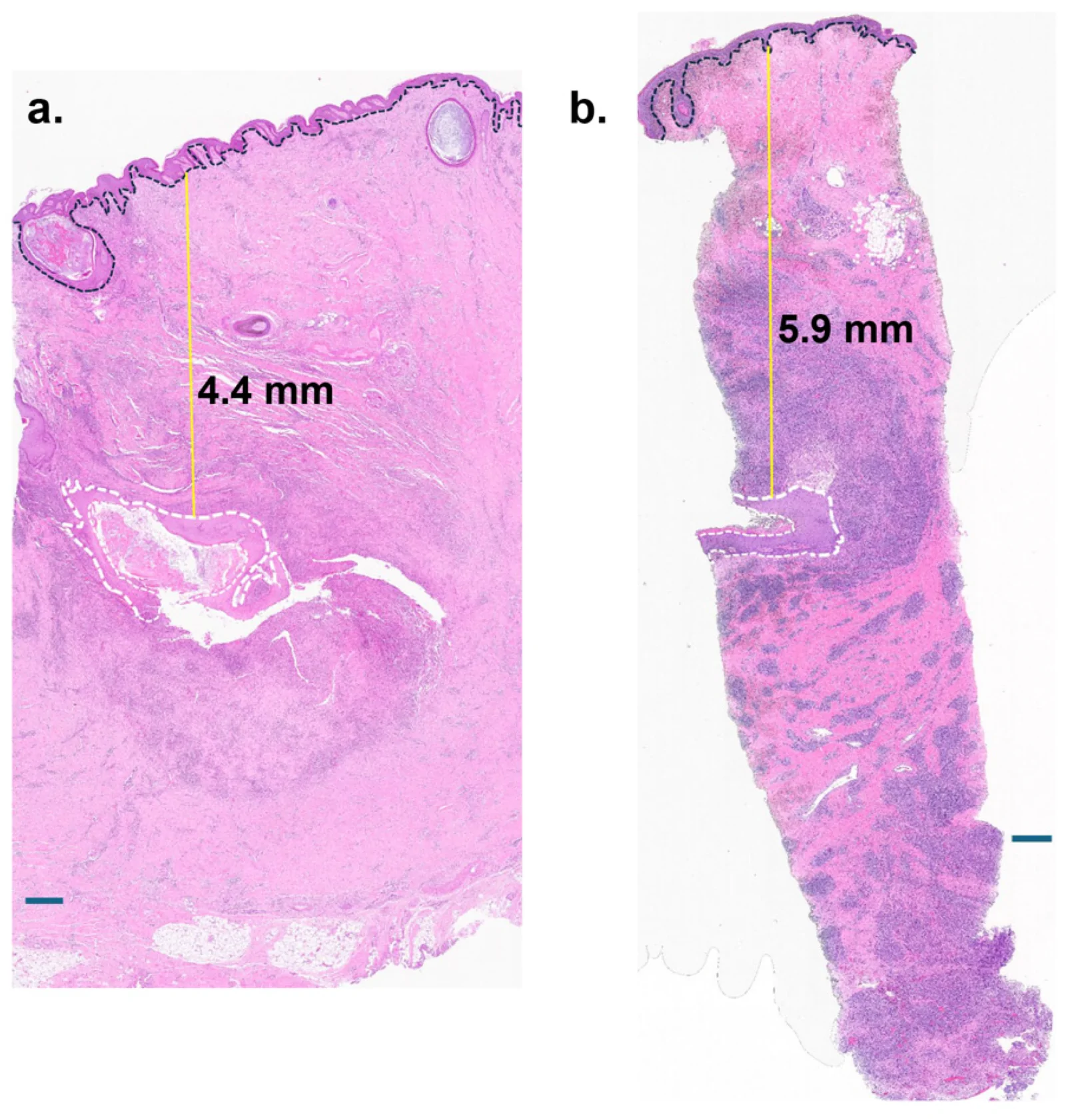
Representative histology of HS tissue excised with SCLM. (**a**) H&E of the tissue post-SCLM intervention, from axillary HS showing robust inflammation throughout the dermis. (**b**) H&E of the tissue excised with SCLM, showcasing infiltrates surrounding the tunnel. The white dashed lines demarcate the tunnel epithelium. The black dashed line demarcates the skin epidermis. The yellow line shows the tunnel depth in the dermis measured from the skin epidermis to the epidermis of tunnel using ImageJ software. Scale bar = 500 µm.

A histomorphometry analysis was performed on the excised tissues from a subset of 15 patients. The tunnel depth was measured from the surface epidermis to the epithelium of the tunnel (Figure 4). The mean depth of epithelialized tunnels was 3.34 mm ± 1.34 mm (range 1.5–5.9 mm). Robust inflammation was associated with the HS tunnels, aligning with previous findings [14], even in the absence of inflammation in the superficial epidermis (Figure 4a).

## 4. Discussion

Tunnels of HS are recognized to be refractory to available medical therapies, particularly in advanced Hurley Stages II–III [3]. Consequently, surgical options are often necessary for management. The initial goal of surgical intervention is to relieve acute discomfort. Incision and drainage are often used for a solitary, painful lesion [7]; however, lesion progression and recurrence at a site is common. Deroofing may be more effective for larger lesions and/or limited sinus tracts [7], since it allows for more complete removal of the roof and margin of inflamed tissue. However, recurrence rates are still quite high, ranging from 10 to 27% [7,15,16].

Full-thickness removal of areas of hidradenitis has become the predominant surgical therapy for larger areas of HS, especially for layered lesions of scar, tunneling or abscess formation. Wide local excision, with or without grafting or other reconstruction, is a standard approach [8,9]. Hospitalization, general anesthesia requirements, extensive surgeries with drains, prolonged healing, resultant scarring, and risk of disease recurrence are consistent concerns surrounding such procedures. Additionally, these interventions may impose a substantial healthcare resource burden due to the use of hospital facilities and personnel, surgical complexity, and management of postoperative complications.

Carbon dioxide laser surgery has been described for several decades. In this series, probe-guided SCLM was used to remove clinically evident lesions followed by sequential identification and excision of sinus tract extensions within the surgical defect during the same operative session. The controlled hemostatic properties of the laser facilitated visualization of occult abscesses and interconnected tracts.

Untreated abscesses and sinus tracts of HS are thought to contain large quantities of inflammatory cytokines, inflammatory cells, and epithelial stem cells [17,18], resulting in an “Invasive Proliferative Gelatinous Mass (IPGM)”, a term coined by William Danby [17]. The histologic analysis of a subset of excised tissue demonstrates that epithelialized tunnels extended an average of 3.34 mm into the dermis, supporting the need for full-thickness removal in advanced disease (Figure 4a,b). In addition, advanced HS tunnels harbor complex anaerobic microbial communities [3,12,19,20]. The thermal effects of CO_2_ laser ablation during the final stages of “carmelization” may contribute to a reduction in the bacterial burden. Although causality cannot be established in this retrospective analysis, the absence of documented post-operative infections across anatomically complex regions is notable and warrants further prospective investigation. Finally, use of the CO_2_ laser in defocused mode provides a more uniform base and may also ablate any remaining epithelial stem cells; indeed, biopsies of the surgical bases failed to identify any remaining cells with epithelial cell markers (Figure 4). One can hypothesize that post-operative recurrent disease observed post-deroofing or -surgical excision may result from persistence of either epidermal stem cells or anaerobic microorganisms.

Our large, multicenter analysis supports that SCLM, with secondary intention healing, provides several features advantageous to the surgical management of HS. In many cases, the procedures were performed using local anesthesia in an outpatient setting, reducing the risks associated with general anesthesia. Secondary intention healing also appears to have resulted in excellent post-operative cosmesis, with minimal risk of wound contractures or reduced range of motion (Figure 1d and Figure 3d), and with minimal risk of recurrence of hypertrophic or keloidal scars.

Across all three sites, nearly 80 patients underwent CO_2_ laser surgery while on a biologic therapy, most commonly adalimumab or infliximab (Table 2). Continuation of systemic therapy during the peri-operative period was not associated with increased post-operative infection in this cohort, in agreement with the literature supporting combined medical and surgical management strategies [21,22].

Documented recurrence of HS at SCLM-treated sites was uncommon in this cohort, with 25 recurrences identified among 1846 treated sites (1.4%). However, the follow-up duration varied substantially, and long-term clinical surveillance was not standardized across patients or treatment centers. Therefore, the observed 1.4% rate should be interpreted as the proportion of treated sites with documented recurrence during the available follow-up rather than as a definitive long-term recurrence rate.

In spite of the benefits noted from this procedure, there are patient and physician challenges that may be experienced. There are no Current Procedural Terminology (CPT) codes that properly describe the technique of SCLM, and insurance approval for such surgery may represent a challenge. Similarly, there are also no CPT codes defining deroofing or partial excision, or if multiple stages are needed for clear margins. Also, the coding for SCLM is problematic, since the procedure is more time consuming than simple excision and requires specialized costly equipment and training. These features are currently not considered in reimbursement to physicians. Adoption of CO_2_ laser surgery may be constrained by equipment requirements, procedural duration, need for trained personnel, and reimbursement structures that do not account for surgical complexity. These practical considerations likely contribute to the relative scarcity of large published series despite its decades of use.

This study has several limitations, including its retrospective design and variable duration and completeness of follow-up. Long-term surveillance was not standardized, and patients without subsequent clinical contact could not be confirmed to be recurrence-free. Consequently, late or unreported recurrences may have been missed, and the observed 1.4% documented recurrence should not be interpreted as a definitive long-term recurrence rate. Additionally, the outcomes were pooled across centers, and although the technique and definitions were standardized, subtle intercenter differences cannot be excluded.

The study period also spans substantial advancements in the medical management of HS. Earlier in the study period, systemic treatment options were more limited, whereas more recent years have seen an increasing use of biologic therapies. As stated, in this cohort a number of patients had previous or current exposure to biologic therapy. The changes in background medical treatment may have influenced the peri-operative disease activity and subsequent outcomes. However, because treatment regimens have varied over time and the outcomes were not analyzed according to the treatment era, the effect of evolving medical management on the observed surgical outcomes cannot be determined from this retrospective study.

In summary, CO_2_ laser marsupialization in this large multicenter cohort demonstrated low recurrence and absence of documented post-operative infections across anatomically challenging regions. SCLM should be actively considered for surgical management of advanced HS that is characterized by increased numbers of nodules, abscesses, and tunnels. While not a novel technology, this approach provides a reproducible surgical option for advanced HS and merits consideration as part of comprehensive treatment strategy. Future studies supporting the mechanism of action of SCLM and controlled long-term follow-ups addressing disease recurrence will confirm the outcomes, eventually leading to broader adoption of this procedure.

## Figures and Tables

**Table 1:** Demographics of HS patients treated with staged CO_2_ laser marsupialization.

	Total (#)
Across Three Treatment Centers *	
Patients	527
Male	161 (30.5%)
Female	366 (69.3%)
Average Age (yrs) +/− SD	35.4 +/− 12.0
Average BMI +/− SD	31.5 +/− 7.0
Average Duration of HS at 1st Encounter (years)	7.5
Ethnicity	
African American	111
Caucasian	323
Hispanic	63
Asian	19
Other/Not Identified	11
Hurley Stage	
Stage I	6 (1.1%)
Stage II	139 (26.3%)
Stage III	383 (72.5%)
Average	2.6
Comorbidities/Co-Influences	
Family History of HS	172
Tobacco Use	119
Diabetes	45
Depression	64
Acne	155
Pilonidal Cyst	137
Pilonidal Cyst & Fam History of HS	87
Folliculitis Decalvans	33
Previous or Current Treatment with Biologic Agent	101

* Treatment Center 1: Hidradenitis Suppurativa Institute, Cleveland, OH, USA; treatment Center 2: Resnik Skin Institute, Miami, FL, USA; treatment Center 3: Center for Laser Surgery, Kaiser Permanente, Union City, CA, USA. SD = standard deviation.

**Table 2:** Profile of HS patients treated with staged CO_2_ laser marsupialization.

	Total (#)
Across Three Treatment Centers *	
Patients	527
Surgical Sessions	992
Local Anesthesia (# patients)	461
General Anesthesia (# patients)	92
Concomitant Treatment with Biologic Agent at the Time of Surgery	
Adalimumab	60
Infliximab	19
Adalimumab + Infliximab	1
Adalimumab + Infliximab + Secukinumab	1
Infliximab + Anakinra	1
Increased Inflammatory Markers	58
Total Areas Treated	1846
Bilateral Axilla	498
R Axilla	135
L Axilla	138
Legs	121
R Thigh	33
L Thigh	39
Breast/Inframammary	44
R Inframammary	7
L Inframammary	7
Chest Wall	62
Abdomen/Pannus	86
Suprapubic	27
Groin/Vulva	372
R Inguinal Fold	78
L Inguinal Fold	82
R Labium Majorum	6
L Labium Majorum	4
Sacrum	5
Pilonidal	20
Buttocks	201
Perirectal/Perianal	54
Scrotum	47
Neck (anterior & posterior)	19
Face	2
Scalp	11
Ear	2
Arms	29
Umbilicus	2
Recurrences (#)	25
Rate of Recurrence (%) (# recurrences/# total areas treated)	1.40

* Treatment Center 1: Hidradenitis Suppurativa Institute, Cleveland, OH, USA; treatment Center 2: Resnik Skin Institute, Miami, FL, USA; treatment Center 3: Center for Laser Surgery, Kaiser Permanente, Union City, CA, USA.

## Data Availability

The data that support the findings of this study are presented within the article. Further inquiries can be directed to the corresponding author.

## Abbreviations

The following abbreviations are used in this manuscript:

SCLM	Staged carbon dioxide laser marsupialization
HS	Hidradenitis suppurativa
CO_2_	Carbon dioxide
CPT	Current procedural terminology
